# Non-Invasive Optical Sensor Based Approaches for Monitoring Virus Culture to Minimize BSL3 Laboratory Entry

**DOI:** 10.3390/s150714864

**Published:** 2015-06-24

**Authors:** Viswanath Ragupathy, Mohan Kumar Hayuri Giri Setty, Yordan Kostov, Xudong Ge, Shaunak Uplekar, Indira Hewlett, Govind Rao

**Affiliations:** 1 LMV/DETTD/OBRR/CBER/FDA, Silver Spring, MD 20993, USA; E-Mails: viswanath.ragupathy@fda.hhs.gov (V.R.); Mohan.Haleyurgirisetty@fda.hhs.gov (M.K.H.G.S.); 2 Center for Advanced Sensor Technology and Department of Chemical, Biochemical and Environmental Engineering, University of Maryland, Baltimore County, MD 21250, USA; E-Mails: kostov@umbc.edu (Y.K.); xge1@umbc.edu (X.G.); shaunak1@umbc.edu (S.U.)

**Keywords:** virus cell culture, BSL3 use, monitoring, optical sensor

## Abstract

High titers of infectious viruses for vaccine and diagnostic reference panel development are made by infecting susceptible mammalian cells. Laboratory procedures are strictly performed in a Bio-Safety Level-3 (BSL3) laboratory and each entry and exit involves the use of  disposable Personnel Protective Equipment (PPE) to observe cell culture conditions. Routine PPE use involves significant recurring costs. Alternative non-invasive optical sensor based approaches to remotely monitor cell culture may provide a promising and cost effective approach to monitor infectious virus cultures resulting in lower disruption and costs. We report here the monitoring of high titer cultures of Human Immunodeficiency Virus-1 (HIV-1) and Herpes Simplex Virus-2 (HSV-2) remotely with the use of optical oxygen sensors aseptically placed inside the cell culture vessel. The replacement of culture media for cell and virus propagation and virus load monitoring was effectively performed using this fluorescent sensor and resulted in half the number of visits to the BSL3 lab (five versus ten).

## 1. Introduction

Human Immunodeficiency Virus (HIV) is transmitted by four different routes, including blood transfusion, sexual contact [1] intravenous drug use and mother to child transmission resulting in the destruction of immune cells (CD4^+^ T cells and development of Acquired Immune Deficiency Syndrome (AIDS) [2]. The rate of disease progression is affected by a number of factors and co-infections, among them a co-infection with Herpes Simplex Virus-2 (HSV-2) being commonly seen with sexual transmission of HIV. HSV-2 is a causative agent of genital herpes in humans. This virus also infects cells of the immune system but does not cause cell death unlike HIV [3]. In an infected human, these viruses can co-exist and serve as co-factors to enhance disease morbidity and mortality. The identification of these virus infections using sensitive diagnostic assays is facilitated by having well characterized reference virus preparations to optimize and validate the assays. Similarly, efforts towards disease prevention through vaccine development may be greatly enhanced by the availability of large scale, high titer virus preparations. Therefore, efficient methods to prepare large-scale cultures are necessary for the development of panels to evaluate diagnostics or the manufacturing of candidate vaccines and new drugs [4].

Traditional methods of propagating these viruses in culture are virus isolation and use of mammalian cell co-cultures carried out in Bio-Safety Level-2 or 3 (BSL-2 or 3) laboratories. These procedures involve costs related to consumables needed to monitor cultures [4]. *In vitro* small scale laboratory culture can be achieved by infecting primary Peripheral Blood Mononuclear Cells (PBMC) and T cell lines in a BSL-3 laboratory in a CO2 incubator at 37 °C, over a period of time. Traditionally, HIV or HSV-2 cultures were monitored for virus growth at ~7 day intervals [3,5]. If there is a progressive infection at each interval, culture flasks are replenished with PBMC cells. In the case of overgrown uninfected controls, 60% of cells are removed, the media changed and cultures are monitored periodically for cell viability. Each entry into a BSL2-3 lab involves extensive use of consumables. Typically, culturing HIV-1 for 21 days involves a minimum of ten entries into BSL-3. For each entry, complete use of personnel protective wear is mandatory. Alternate culture monitoring methods could reduce the number of visits to the BSL-3 laboratory. Novel online real-time monitoring methods are needed that are less labor-intensive and cost effective. Several engineered methods are available to monitor cell proliferation [6,7,8], but these methods are useful for one time individual operation. A study published in 2008 [9] by directly measuring dissolved oxygen and pH provides a reliable approach to long-term online monitoring. As the cell grows, the biomass increases with lactic acid production and steady decrease of pH of the growth medium. In addition, dissolved oxygen and pH levels are the indicators of the onset of programed cell death (apoptosis) [10]. Measuring these two biological indicators may provide an alternative approach for in vitro cell culture systems. Recently, non-invasive dissolved oxygen monitoring using a fluorescence-based patch developed to monitor small scale cultures has been shown to be a promising approach [11,12] to be used for virus cultures. The patch has a fluorescent dye immobilized in a polymer matrix. They are affixed inside the culture flask with the electronics required for measurement being placed outside. The device is minimally invasive, except for the patch, which is very thin. The rest of the system is located outside and the measurement is made non-invasively through the transparent vessel wall. These sensors can be useful in scaling up from lab to bench scale and beyond and in developing small-scale plat- forms for process optimization. However, despite the potential advantages of implementing this technology, it has not been widely adopted for use in industry and academia as there is resistance to adopt such new practices unless there is compelling evidence for their utility. In our pilot study we used the CEM-ss T-cell line (Human CD4positive lymphoblastoid cell line obtained from NIH AIDS Reagent Program (Cat. No.: 776) to monitor infectivity of HIV-1 and HSV-2 and correlate it with HIV-1 viral load measurement in terms of p24 ng/mL using the oxygen sensor. This is a proof of concept study using two unrelated viruses (differences in pathogenesis). If this approach is feasible, alternate *in vitro* cell culture monitoring could be adopted for other pathogens or select agents. 

**Figure 1:**
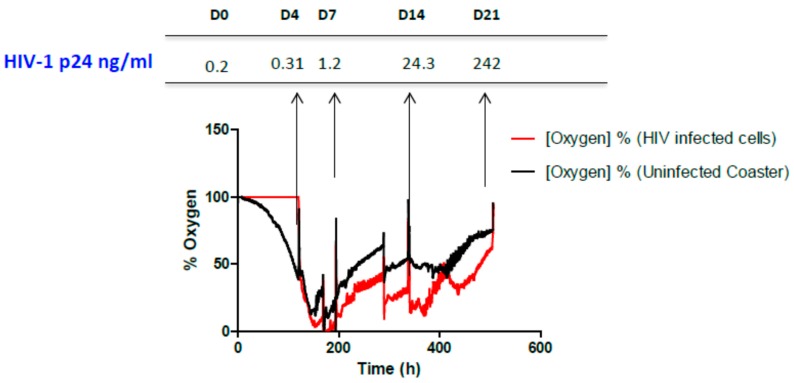
HIV culture monitoring through oxygen sensor and correlation with HIV-1 viral load (p24 ng/mL) quantification. Each spike in the figure indicates a BSL-3 visit was made to replenish cells and collect an aliquot of culture supernatant for HIV titer determination.

In our present work, potential respiration differences between flasks containing infected and un-infected cultures were compared in terms of continuous monitoring using the fluorescent dye patch. The aim was to evaluate whether this approach could help determine when to feed the flask with cells or subculture them. CEM-ss cells were infected as described in methods and the dissolved oxygen in the flask was continuously monitored for ~400 h (Figure 1). Sensor sampling was performed every 30 min. 

**Figure 2:**
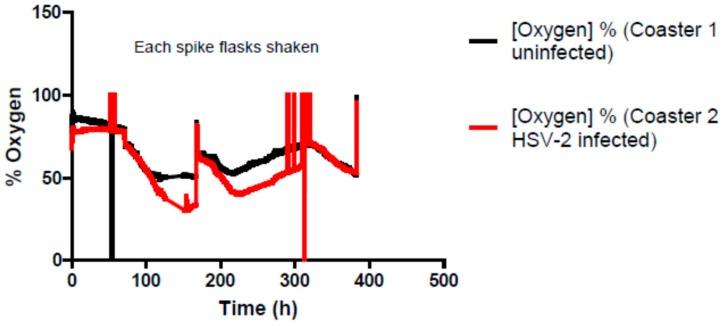
HSV-2 infection monitoring of sensor patches. Each spike in the figure indicates a BSL-3 visit was made to replenish cells and collect an aliquot of culture supernatant for HSV-2 titer determination.

The sensor was also evaluated for its correlation with HIV-1 viral load in terms of HIV-1 p24 levels. Before placing culture flasks over the electronic sensor, an aliquot of culture supernatant was collected that served as the day 0 sample. This experiment was carried out for 21 days and supernatants were collected at seven day intervals. HIV replication increased with time and a reduction in respiration of cells was observed. As the equilibrium was established by feeding cells at day 14 and 21, a rise in oxygen saturation was observed (Figure 1) with positive rate change. In contrast, in the uninfected flask, oxygen saturation decreased similar to the infected flask, likely due to masking of sensor patches with over grown cells. In order to keep the equilibrium constant, the culture medium was replenished at intervals when they achieved levels of oxygen below 50% or virus titers as high as 242 ngs/mL were observed at day 21. Finally, the sensor patch was also evaluated with a virus that infects its susceptible cells but does not cause its destruction upon replication. One such example is HSV-2. Productive infection is noted from a Taqman assay comparing day 4 (ct 28.6) and day 14 (ct 22.6) virus load (see Supplementary material). Similar to HIV, after a certain period of time, there was a decline in the oxygen concentration obtained with both infected and uninfected cells (Figure 2). Changes in the pH levels of the culture medium were also observed, indicating that cells were over grown and required a subculture. After the subculture, the cells again attained the maximum oxygen percentage. 

The advantage of optical sensors is miniaturization and high sensitivity for monitoring dissolved oxygen. Despite these advantages, there were concerns about whether the dye might leach and induce cell toxicity, as it was previously shown that there were no negative effects in cell physiology at the transcript level [13]. In our present study, it was found that cell cultures could be carried out for up to 21 days, which is the typical time period for culturing HIV viruses [14]. The sensor patch was able to monitor changes in oxygen levels for 21 days confirming the feasibility of this approach for monitoring virus-infected cell cultures and the BSL-3 visits were reduced to half (five visits) compared to the traditional approach.

## 2. Materials and Methods

**Figure 3:**
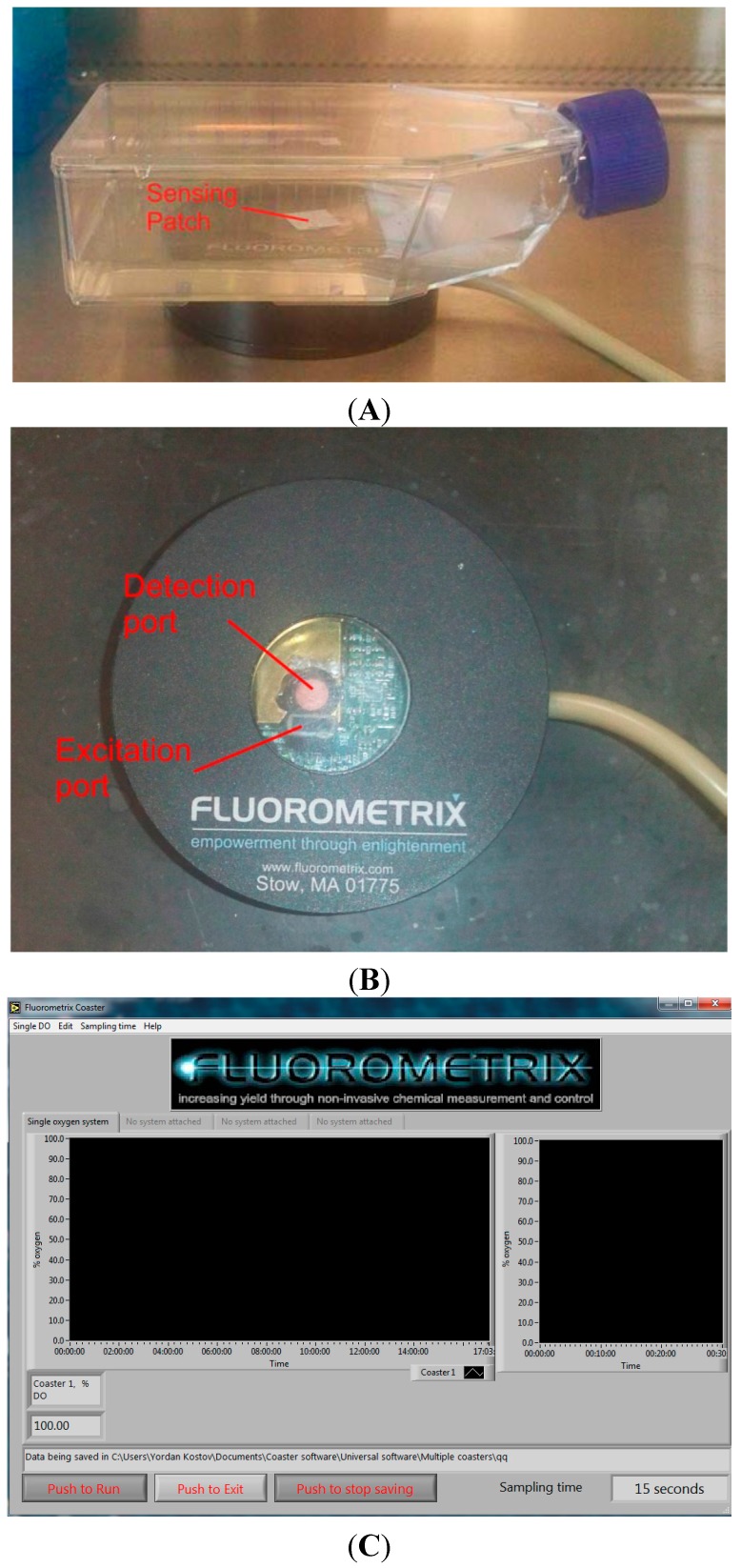
(**A**) Fluorometrix Coasters; (**B**) Fluorometrix Coasters monitoring culture flask; (**C**) Fluorometrix data recording system.

The sensor (CellPhase^TM^, Fluorometrix, currently available from Scientific Industries, Inc. New York, NY, USA) consists of an in dwelling fluorescent oxygen-sensing patch, external optoelectronics and software for user interfaces. A sterile optical patch was placed aseptically in T-75 cm^2^ flasks (Figure 3A). The patch changes its fluorescence lifetime in response to the changes in oxygen partial pressure. The changes in fluorescence are detected by optoelectronics (Figure 3B). It interrogates the patch with modulated excitation at 470 nm and detects emission at 610 nm. The digitized fluorescent signals are sent to a laptop located outside the BSL3. The fluorescence decay rate is calculated there and converted into an oxygen concentration using manufacturer-supplied calibration codes. The user interface (Figure 3C) displays both the instantaneous value of the dissolved oxygen as well as the time course of the oxygen concentration. 

At present CEM-ss, cells that support the infection of HIV-1 or HSV-2, were used as per the standard laboratory procedures (see Supplementary material). Briefly, cells were infected with equal quantities of these viruses, and dissolved oxygen was monitored every 30 min over 21 days. At days 7, 14, and 21 an aliquot of culture supernatant was removed for HIV-1 p24 ELISA and HSV-2 Taqman measurements (viral load).

## 3. Conclusions

In summary, we demonstrated a novel application using non-invasive optical sensor patch based approaches for monitoring oxygen levels in small or large scale production of infectious virions. This approach may reduce operational costs and labor by virtue of its ability to provide remote metabolic monitoring. Results from our pilot studies may have implications for virus production for vaccines or diagnostic applications.

## Supplementary Materials

Supplementary materials can be accessed at: http://www.mdpi.com/1424-8220/15/7/14864/s1.
