# Supplementary material for: Non-Invasive Optical Sensor Based Approaches for Monitoring Virus Culture to Minimize BSL3 Laboratory Entry
Source: Sensors (Basel). 2015 Jun 24;15(7):14864–70. doi: 10.3390/s150714864 (PMC4541811; doi:10.3390/s150714864)
Supplement: Supplementary file 1 [file sensors-15-14864-s001.pdf]

OPEN ACCESS

sensors

ISSN 1424-8220

www.mdpi.com/journal/sensors

Supplementary Information

Non-Invasive Optical Sensor Based Approaches for Monitoring Virus Culture to Minimize BSL3 Laboratory Entry. *Sensors* 2015, *15*, 14864-14870

Viswanath Ragupathy [1], Mohan Kumar Hayuri Giri Setty [1], Yordan Kostov [2], Xudong Ge [2], Shaunak Uplekar [2], Indira Hewlett [1,*] and Govind Rao [2,*]

[1] LMV/DETTD/OBRR/CBER/FDA, Silver Spring, MD 20993, USA; E-Mails: viswanath.ragupathy@fda.hhs.gov (V.R.); Mohan.Haleyurgirisetty@fda.hhs.gov (M.K.H.G.S.)

[2] Center for Advanced Sensor Technology and Department of Chemical, Biochemical and Environmental Engineering, University of Maryland, Baltimore County, MD 21250, USA; E-Mails: kostov@umbc.edu (Y.K.); xge1@umbc.edu (X.G.); shaunak1@umbc.edu (S.U.)

* Authors to whom correspondence should be addressed; E-Mails: Indira.Hewlett@fda.hhs.gov (I.H.); grao@umbc.edu (G.R.); Tel.: +240-402-9587 (I.H.); +410-455-3415 (G.R.).

Academic Editor: Stephane Evoy

Received: 24 March 2015 / Accepted: 15 June 2015 / Published:

1. Estimation of HSV-2 Virus Replication

Lower the average cycle threshold (ct) higher the virus load. As seen from above table at Day 14 ct of 22.6 indicate active virus growth as compared to day 4 (Table S1).

Table S1. Estimation of HSV-2 virus replication.

	**HSV2 Taqman**								
	**Day 4**		**Average Ct**	**Day 7**		**Average Ct**	**Day 14**		**Average Ct**
	**Replicate 1**	**Replicate 2**		**Replicate 1**	**Replicate 2**		**Replicate 1**	**Replicate 2**	
Uninfected	0	0	0	0	0	0	0	0	0
Control	0	0	0	0	0	0	0	0	0
HSV-2 Infection	28.594	28.676	28.635	28.197	29.405	28.801	22.676	22.610	22.643

2. Cell Culture and Infectivity of HIV or HSV-2

CEMss cells were received from the NIH AIDS Reagent program and cultured in RPMI 1640 supplemented with 10% FBS, 2 mM glutamine, 100 units/mL of penicillin, and 100 units/mL streptomycin. Prior to infectivity experiments, the cells were prepared at a concentration of 1 million/mL (5 million total) in serum free media. The HIV-1MN lab adapted strain was used for infectivity at a concentration of 5ng/mL of HIV-1 p24 antigen. After 2 h of infection at 37 °C, 5% CO_2, cells were washed twice with 10 mL of 1× Phosphate Buffer Saline (PBS) and the cell pellet was resuspended in 40 mL of culture media (RPMI with 10% FBS). Culture media containing cells were transferred to a T-75 cm^2 flask containing the fluorescent dye patch. Similar concentrations of uninfected cells were prepared and transferred to a T-75 cm^2 flasks containing fluorescent dye patch.

We had previously shown that HSV-2 can infect the CEMss cell line (unpublished). Therefore, cells were prepared according to methods used for HIV infection and 5pfu (plaque forming units) of HSV-2 was used for infectivity studies. HIV and HSV-2 infection was performed in two separate experiments with a control flask containing no viruses. Dissolved oxygen was monitored every 30 min over 21 days. At days 7, 14, and 21 an aliquot of culture supernatant was removed for HIV-1 p24 measurement.

3. Determination of Infectivity Titers

3.1. HIV

1 mL of culture supernatant was harvested at 4, 7, 14 and 21 days post infection for p24 antigen measurements in duplicate. HIV-1 p24 quantification was performed using the Perkin-Elmer HIV-1 p24 Elisa Kit, Cat No: NEK050B001. All procedures were followed as per the manufacturer instructions. Because this kit was optimized for quantification in a narrow range 12.5–200 pg/mL for HIV-1 p24, culture supernatants were diluted in culture media to 1:10, 1:100 and 1:1000 for quantification. Data were analyzed from excel spread sheet with graph pad software v5.0.

3.2. HSV-2

1 mL of culture supernatant was harvested at 4, 7 and 14 days post infection for HSV-2 taqman assay in duplicate. Briefly Nucleic acids were extracted according to manufactures instructions of QIAGEN (Cat No: 57704) and determined using in-house real time PCR. A 118-nucleotide segment of the gB region was amplified by the use of primers described in Lilly Namvar *et al.* (J. Clin. Microbiol., 2005).

© 2015 by the authors; licensee MDPI, Basel, Switzerland. This article is an open access article distributed under the terms and conditions of the Creative Commons Attribution license (http://creativecommons.org/licenses/by/4.0/).
